# Biopsy-proven first dose of oxaliplatin-induced acute tubular necrosis leading to end-stage renal failure: a case report

**DOI:** 10.1186/s12882-023-03116-2

**Published:** 2023-03-28

**Authors:** Yu Soma, Taiichi Kawabe, Daiyu Kitaji, Kaoru Hoshino, Sumire Sunohara, Takehisa Iwano, Naomi Kawano

**Affiliations:** 1 Department of Nephrology, Yokohama Minami Kyousai Hospital, Yokohama, Japan; 2 Department of Digestive surgery, Yokohama Minami Kyousai Hospital, Yokohama, Japan; 3 Department of Pathology, Yokohama Minami Kyousai Hospital, Yokohama, Japan

**Keywords:** Oxaliplatin, Acute kidney injury, Acute tubular injury, Maintenance dialysis

## Abstract

**Background:**

Oxaliplatin is an anticancer therapy for pancreatic, gastric, and colorectal cancers. It is also used in patients with carcinomas of unknown primary sites. Oxaliplatin is associated with less frequent renal dysfunction than other conventional platinum-based drugs such as cisplatin. Albeit, there have been several reports of acute kidney injury with frequent use. In all cases, renal dysfunction was temporary and did not require maintenance dialysis. There have been no previous reports of irreversible renal dysfunction after a single dose of oxaliplatin.

**Case presentation:**

Previous reports of oxaliplatin-induced renal injury occurred after patients received multiples doses. In this study, a 75-year-old male with unknown primary cancer and underlying chronic kidney disease developed acute renal failure after receiving the first dose of oxaliplatin. Suspected of having drug-induced renal failure through an immunological mechanism, the patient was treated with steroids; however, treatment was ineffective. Renal biopsy ruled out interstitial nephritis and revealed acute tubular necrosis. Renal failure was irreversible, and the patient subsequently required maintenance hemodialysis.

**Conclusions:**

We provide the first report of pathology-confirmed acute tubular necrosis after the first dose of oxaliplatin which led to irreversible renal dysfunction and maintenance dialysis.

## Background

Oxaliplatin is an anticancer therapy for pancreatic, gastric, and colorectal cancers [1]. It is also used in patients with carcinomas of unknown primary sites [2]. Oxaliplatin is associated with less frequent renal dysfunction than other conventional platinum-based drugs such as cisplatin [3]. Despite that, there have been reports of acute tubular necrosis (ATN) with more frequent use [4–7]. In all cases, renal dysfunction was temporary and did not require maintenance dialysis. In this report, we describe the first case wherein renal biopsy showed ATN despite a single oxaliplatin administration; additionally, renal dysfunction was irreversible, leading to maintenance dialysis.

## Case presentation

A 75-year-old male underwent surgeries for ascending colon cancer and liver metastasis in the previous 8 and 6 years, respectively. Thereafter, the patient remained recurrence-free. He was then followed-up regularly by a gastrointestinal surgeon. No other underlying diseases such as hypertension and diabetes were observed; no regular medications were taken. Follow-up computed tomography (CT) 3 months prior to hospitalization revealed ascites and multiple peritoneal seeding. Transgastric biopsy of the peritoneal nodules was performed using endoscopic ultrasound-guided puncture aspiration. Immunostaining was negative for colon cancer metastasis, and unknown primary cancer was diagnosed. During pre-treatment assessment, his laboratory workup revealed chronic renal dysfunction equivalent to chronic kidney disease (CKD) G3bA1 (serum creatinine [sCr] level 1.3–1.6 mg/dL) without abnormal urinalysis results. Therefore, cisplatin was avoided in favor of tegafur/gimerasil/oterasil (S-1) plus oxaliplatin (SOX regimen) as the anticancer regimen of choice. To account for renal dysfunction, both S-1 and oxaliplatin were administered at reduced doses (80 mg/day of S-1 instead of the normal dose at 120 mg/day and 100 mg/m^2^ of oxaliplatin instead of 130 mg/m^2^). Three weeks after the first SOX infusion, the second dose was scheduled. The sCr level at that time rose to 3.17 mg/dL, and acute kidney injury (AKI) was diagnosed. Therefore, the second dose of SOX was not administered. Two weeks later, renal dysfunction progressed and the patient was urgently hospitalized. Prominent edema was observed in both lower legs. The patient had abdominal distention due to ascites accumulation, but no abdominal symptoms. There were no other abnormalities in physical examination. Blood tests showed a normal white blood cell count (7100/µL), low hemoglobin level (7.8 g/dL), and normal platelet count (160,000/µL). Schistocytes were not found in peripheral blood smear; sCr level progressed to 5.02 mg/dL. C-reactive protein level was elevated (19.7 mg/dL) and remained persistently high (10–20 mg/dL). Lactate dehydrogenase level was also elevated (740 U/L). Result of infectious workup was negative, therefore, high C-reactive protein level was considered. Antineutrophil cytoplasmic, anti-nuclear, and anti-glomerular basement membrane antibodies were absent. Von Willebrand factor-cleaving protease (ADAMS13) level was normal. Urine N-acetyl-β-D-glucosaminidase levels were slightly high (570 µg/L, reference value < 289 µg/L), while N-acetylglucosaminidase level was mildly elevated (82.6 IU/L, reference value 0.7–11.2 IU/L). Urine microscopy findings revealed granular casts and glomerular hematuria was absent. Urinary protein excretion was approximately 1 g/day. The drug-induced lymphocyte stimulation test (DLST) was positive for oxaliplatin and negative for S-1. Ascitic fluid was confirmed by CT scan, which showed no renal atrophy, postrenal acute renal injury, and an intravesical pressure of 5 mmHg (normal value 5–7 mmHg), negating abdominal compartment syndrome. Drug-induced renal dysfunction was suspected; however, as renal dysfunction did not improve after drug withdrawal, methylprednisolone (mPSL) pulse therapy (500 mg/dose daily for 3 days) was initiated on day 3 of hospitalization, based on the belief that immunological mechanisms were involved. After the 3-day course, mPSL was changed to prednisolone (PSL) 50 mg/day (0.8 mg/kg/day). Renal biopsy was performed on the day 8 of hospitalization.

**Fig. 1:**
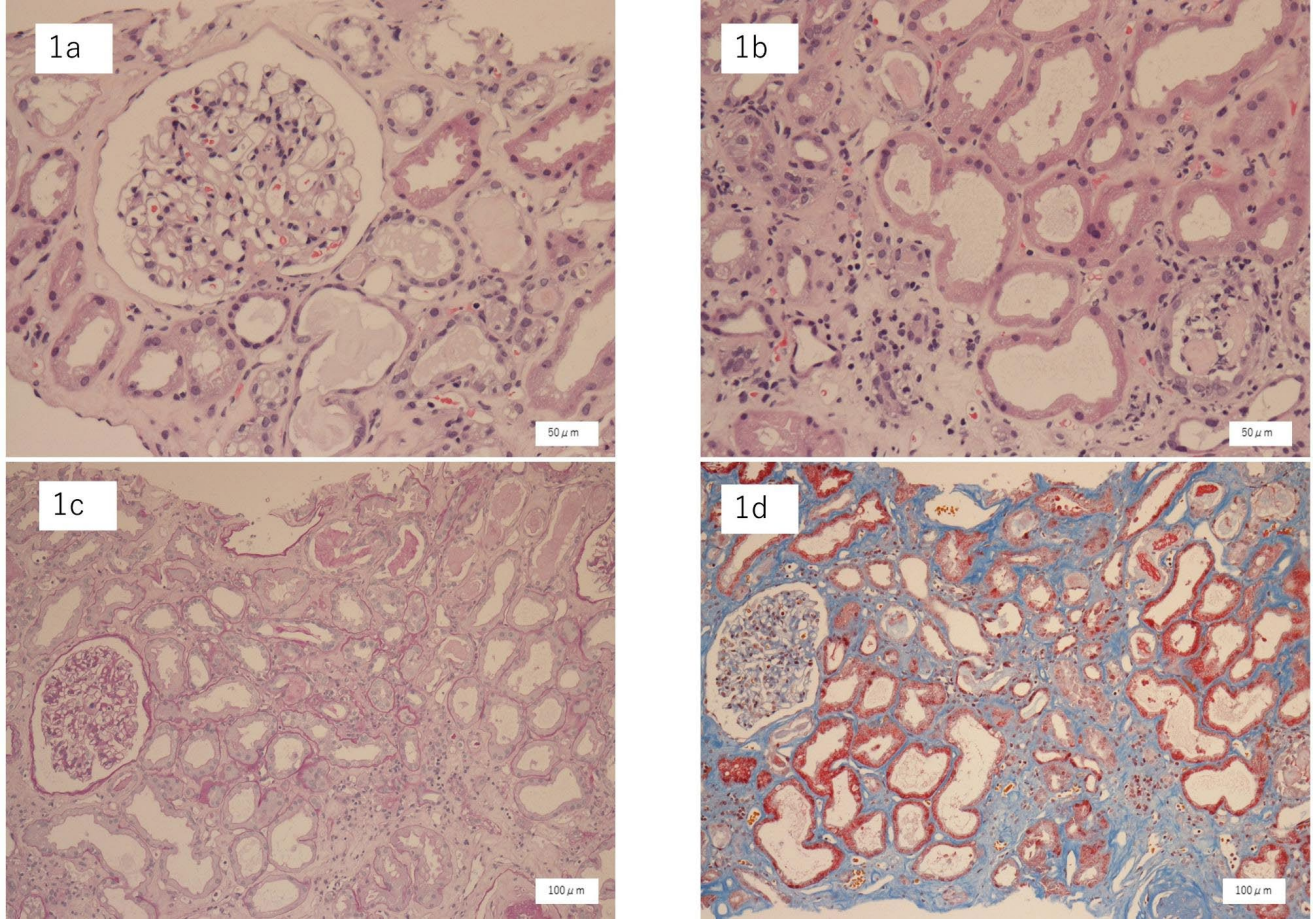
Renal biopsy. (**a, b**) Hematoxylin and eosin staining, x 400. Five glomeruli showed global sclerosis. The other glomeruli were normal. The tubules showed diffuse degenerative findings, including severe atrophy, obscuration of the print border, and flattening of the epithelium. (**c**) Periodic acid–Schiff staining, x 400. Lymphocyte infiltration in the interstitium and tubules was unremarkable. (**d**) Elastica Masson staining, x 400. Interstitial fibrosis in the interstitium and tubules was unremarkable

Thirty-five glomeruli were collected, of which 5 had global sclerosis. A mild increase in mesangium substrate was observed in several glomeruli; no other abnormal findings were observed in the other glomeruli (Fig. 1a). The tubules showed diffuse degenerative findings, including severe atrophy, obscuration of the print border, and flattening of the epithelium (Fig. 1b). Lymphocyte infiltration in the interstitium and tubules was unremarkable (Fig. 1c) and interstitial fibrosis was mild (Fig. 1d). Vasculitis or thrombus formation was not observed. In the glomeruli, the IgG, IgA, C1q, and C3 assays showed negative results, implying a lack of glomerular involvement.

Pathologically, the findings indicated acute tubular necrosis. Considering the pathological findings and DLST results, a diagnosis of acute tubular injury due to oxaliplatin was made. Renal function continued to deteriorate with a sCr of 7.77 mg/dl on day 11 of admission. The patient was oliguric, had prominent generalized edema, and was started on hemodialysis. Thereafter, the patient did not show any improvement in renal dysfunction and was placed on maintenance hemodialysis. PSL was tapered and terminated.

## Discussion and conclusions

Reports of oxaliplatin-induced AKI are rare. Temporary dialysis may be required in some patients however, when ATN was the sole pathology, renal dysfunction improved with conservative treatment [4–7]. In these reports, oxaliplatin was administered multiple times. As shown in Table 1, this case is unlikely to have ATN due to the cumulative doses of oxaliplatin as in previous reports [4–7]. In our patient, both serological and pathological thrombotic microangiopathy (TMA) also was ruled out. There have been reports of AKI in patients treated with S-1 [8], however, in this case, only oxaliplatin was determined to be the causative agent according to DLST results. DLST measures the proliferation of T cells induced by an agent in vitro. It leads to suspicion of previous sensitization in vivo. There are case series of patients with positive DLST and diagnosis of drug-induced hypersensitivity nephritis [9]. DLST is also considered a potentially useful test for drug hypersensitivity, including drug-induced hypersensitivity nephritis [10]. Although there have been reports of oxaliplatin-induced TMA, it was ruled out serologically or pathologically in our patient [11]. Moreover, there have been reports of oxaliplatin-induced acute interstitial nephritis wherein PSL was administered prior to obtaining pathology-confirmed diagnosis due to PSL being necessary in such cases [12, 13]. In our patient, as shown in Fig. 1b and d, the tubules showed diffuse degenerative findings, including severe atrophy, obscuration of printed borders, and flattening of the epithelium. Conversely, lymphocytic infiltration in the interstitium and tubules was unremarkable. Acute interstitial nephritis (AIN) was ruled out due to the absence of marked lymphocytic infiltration into the interstitium. In addition, AIN is often associated with systemic symptoms and skin rashes [14]. However, in the present case, no such features were observed. PSL was considered unnecessary and was tapered off. Given conservative treatment did not improve renal dysfunction as expected, the patient was placed on maintenance hemodialysis. AIN was ruled out based on pathological results and clinical course, which included no systemic symptoms and an ineffective PSL. Increased risk of end-stage renal disease (ESRD) has been reported with AKI [15]. The patient had chronic renal dysfunction equivalent to CKD G3b in the base, which may have led to an irreversible transition to ESRD despite AKI with ATN.

**Table 1:** Reported cases of biopsy-proven oxaliplatin-induced acute tubular necrosis (n = 5)

References	Age/sex	Type of tumor	Type of chemotherapy	Total frequency of oxaliplatin received	Oxaliplatin total dosage	Base sCr	Peak sCr	Dialysis dependence	immunosuppressive drug	Renal outcome* (Final sCr)
[4]	57/M	Colon	FOLFOX	17th	1150 mg/m2	Unknown	7.3	None	None	CR (Details unknown)
[5]	65/M	Colon	FOLFOX	5th	470 mg	0.77	12.2	Yes	None	PR (sCr 1.35 mg/dl)
[6]	67/M	Colon	FOLFOX	13th	Details unknown	0.9 units	12.1	Yes	None	CR (sCr 1.1 mg/dl)
[7]	69/F	Ovarian	Oxaliptatin/gemcitabin	10th	1210 mg	0.8	12.7	Yes	None	CR (sCr 1.0 mg/dl)
Present case	75/M	Unknown primary cancer	SOX	1st	160 mg	1.3–1.6	7.77	Yes	Steroid	End-stage renal disease

*Complete recovery was defined as recovery of renal function to within 0.3 mg/dl from base sCr. Partial recovery was defined as recovery of renal function that worsened to > 0.3 mg/dl from the base sCr

CR: complete recovery, PR: partial recovery, M: male, F: female, FOLFOX: 5-fluorouracil (5-FU)/l-leucovorin/oxaliplatin, sCr: serum creatinine (mg/dL), SOX: tegafur/gimerasil/oterasil (S-1) plus oxaliplatin

As shown in Table 1, in the 4 patients with confirmed ATN and receiving oxaplatin treatment, who are known to have a base sCr less than 1 mg/dL, improvement in renal dysfunction was observed [4–7]. The patient in our case had a base sCr of 1.3–1.6 mg/dL and more severe chronic renal dysfunction than the previous 4 cases, thereby possibly leading to irreversible renal dysfunction.

In conclusion, we provide the first report of pathology-confirmed ATN after the first dose of oxaliplatin which led to irreversible renal dysfunction. This suggests that oxaliplatin may need to be considered as a potential cause of ATN in patients with renal dysfunction despite the absence of cumulative dosing.

## Data Availability

Raw data were generated at Yokohama Minami Kyousai Hospital. Derived data supporting the findings of this study are available from the corresponding author (Yu Soma, sanshine1133@yahoo.co.jp) on request.

## List of abbreviations

AKI: acute kidney injury
ATN: acute tubular necrosis
CT: computed tomography
CKD: chronic kidney disease
S-1: tegafur/gimerasil/oterasil
SOX regimen: (S-1) plus oxaliplatin
sCr: serum creatinine
ADAMS13: Von Willebrand factor-cleaving protease
DLST: drug-induced lymphocyte stimulation test
mPSL: methylprednisolone
PSL: prednisolone
TMA: thrombotic microangiopathy
AIN: acute interstitial nephritis
ESRD: end-stage renal disease
